# Changes in Diet and Anthropometric Parameters in Children and Adolescents with Celiac Disease—One Year of Follow-Up

**DOI:** 10.3390/nu13124306

**Published:** 2021-11-28

**Authors:** Agnieszka Kozioł-Kozakowska, Dominika Salamon, Zofia Grzenda-Adamek, Agnieszka Krawczyk, Mariusz Duplaga, Tomasz Gosiewski, Kinga Kowalska-Duplaga

**Affiliations:** 1 Department of Pediatrics, Institute of Pediatrics, Gastroenterology and Nutrition, Jagiellonian University Medical College, 30-663 Krakow, Poland; agnieszka.koziol-kozakowska@uj.edu.pl; 2 Department of Molecular Medical Microbiology, Chair of Microbiology, Jagiellonian University Medical College, 31-121 Krakow, Poland; dominika.salamon@uj.edu.pl (D.S.); a.krawczyk993@gmail.com (A.K.); tomasz.gosiewski@uj.edu.pl (T.G.); 3 Department of Pediatrics, Gastroenterology and Nutrition, University Children’s Hospital, 30-663 Krakow, Poland; zgrzenda@hotmail.com; 4 Department of Health Promotion and e-Health, Institute of Public Health, Faculty of Health Sciences, Jagiellonian University Medical College, 31-066 Krakow, Poland; mariusz.duplaga@uj.edu.pl

**Keywords:** celiac disease, gluten-free diet, pediatric patients, nutritional status

## Abstract

Celiac disease (CD) may cause numerous nutrient deficiencies that a proper gluten-free diet (GFD) should compensate for. The study group consists of 40 children, aged 8.43 years (SD 3.5), on average, in whom CD was diagnosed on the basis of clinical symptoms, immunological and histopathological results. The patients’ height, weight, diet and biochemical tests were assessed three times: before diagnosis, after six months, and following one year of GFD. After one year, the patients’ weight and height increased but nutritional status (body mass index, BMI percentile) did not change significantly. The children’s diet before diagnosis was similar to that of the general Polish population: insufficient implementation of the dietary norm for energy, fiber, calcium, iodine, iron as well as folic acid, vitamins D, K, and E was observed. Over the year, the GFD of the children with CD did not change significantly for most of the above nutrients, or the changes were not significant for the overall assessment of the diet. Celiac patients following GFD may have a higher risk of iron, calcium and folate deficiencies. These results confirm the need for personalized nutritional education aimed at excluding gluten from the diet, as well as balancing the diet properly, in patients with CD.

## 1. Introduction

Celiac disease (CD) is a chronic, multifactorial autoimmune disease related to the ingestion of gluten that affects the small bowel in genetically predisposed persons (mutations in the human leukocyte antigen (HLA) DQ2 and/or DQ8 haplotype), which results in a wide range of clinical symptoms, both gastrointestinal and extra-gastrointestinal [1,2]. Gluten in one’s diet causes progressive atrophy of the villi in the small intestine resulting in an alteration in the absorption of nutrients; thus, leading to various deficiency states [3]. Although the disease has been known for a long time, it has been given a lot of attention in recent years due to its increasing prevalence. There has been a substantial increase in the numbers of new cases, partly due to better diagnostic tools and thorough screening of individuals considered to be at high risk for the disorder [4]. In Europe, the histologically confirmed prevalence is around 0.6%. and the serological screening of the general population gave the result of 1%; in Poland, it is about 0.8–1% of the total population [5,6]. CD is more common in white populations, especially in western and northern European regions. The basis for the treatment of celiac disease is the strict compliance of a gluten-free diet (GFD), which excludes all products containing gluten such as wheat, barley, rye, and all products derived from these grains, such as starch, flour, bread, pasta, etc. Oats, due to the risk of gluten contamination, are also excluded from the diet.

Patients with newly diagnosed CD often have nutrient deficiencies, especially involving iron, calcium, zinc, folic acid, vitamin D, and other fat-soluble vitamins, as a result of malabsorption following damage to the small intestine mucosa that occurs in celiac disease. Strict adherence to the GFD facilitates regeneration of intestinal villi, enabling proper absorption of nutrients [7]. However, some authors have reported an unbalanced diet in terms of macro and micronutrients in celiac patients and, in this situation, nutritional deficiencies associated with GFD are more so caused by the diet than the disease mechanism [7]. Improperly balanced GFD can make deficiencies worse rather than improve them, which is extremely dangerous for children during their development.

Children with untreated celiac disease were usually shorter than their healthy peers, which is a consequence of nutritional deficiencies [8]. CD individuals also have a higher prevalence of underweight, as well as a lower presence of obesity, as compared to non-celiac peers [8]. However, some patients on a gluten-free diet develop obesity. The incidence of excess body weight in pediatric patients with celiac disease on a gluten-free diet ranges from 9.4% to 21% [9,10]. The increasing prevalence of excess body weight in CD patients can be explained by the global trend towards weight gain in the children general population, but also inappropriate nutritional education of patients and their parents after diagnosis. Unfortunately, there are not many studies on this topic. Our study aimed to assess the change in diet and anthropometric parameters of CD patients during the first year of adhering to the gluten-free diet.

## 2. Material and Methods

### 2.1. Study Design and Population

**Figure 1:**
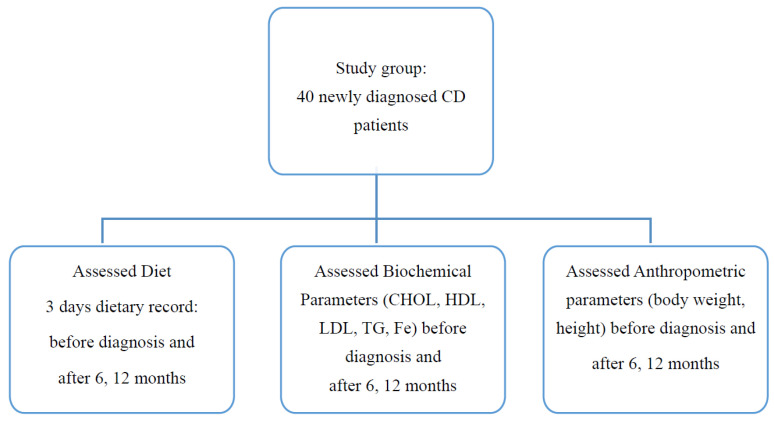
Flowchart of study design.

The study group consists of patients diagnosed and treated in the Department of Pediatrics, Gastroenterology, and Nutrition, University Children’s Hospital in Krakow, in the years 2019–2021. The inclusion criteria for the study were as follows: children and adolescents aged 2–18 years in whom CD was newly diagnosed on the basis of clinical symptoms, results of immunological tests, and histopathological pictures of biopsies taken from the duodenum. When recruiting patients, we used the European Society Paediatric Gastroenterology, Hepatology and Nutrition guidelines in force at the time when the study began [2]. Parents or legal guardians of the children, as well as the patients over 16 years of age, gave consent to participate in the study. The exclusion criteria were: age below 2 and above 18 years; confirmed infections or chronic diseases of the gastrointestinal tract (e.g., inflammatory bowel disease); obesity, thyroid diseases; neoplastic diseases (especially of the gastrointestinal tract); immunodeficiencies; lack of consent of the patient and/or legal guardian for participation in the study. In all patients, Immunoglobulin A (IgA) deficiency was excluded, the titer of antibodies to tissue transglutaminase in IgA class (TTG IgA) and the coeliac-related genotype HLA DQ2/DQ8 (CeliacStrip; Operon) were assessed. An enzyme-linked immunosorbent assay test (ELISA) was used to determine antibody titers, with levels above 20 RU/mL considered as abnormal. All laboratory tests were performed in a hospital laboratory in accordance with Good Laboratory Practice (GLP) rules and using tests with In Vitro Diagnostics/Food and Drug Administration (IVD/FDA) certificates intended for routine medical diagnosis and being part of the clinical protocol in specialized laboratories of the University Children’s Hospital in Krakow. All patients underwent gastroscopy with specimen collection, obtained from both the duodenum and the duodenal bulb, for histopathological evaluation. The degree of villous atrophy was determined using the Marsh–Oberhuber classification [11]. Once the diagnosis was confirmed, the patients and their parents were instructed to follow a gluten-free diet. At 3 measurement points, i.e., before diagnosis, after six months and after one year on the diet, the patients were assessed for height and weight, laboratory tests were also taken, and their diet was evaluated (Figure 1).

### 2.2. Anthropometric Parameters

Body weight and height were measured to the nearest 0.1 kg and 0.1 cm, respectively, using a stadiometer and a balance scale (SECA, Hamburg, Germany). As the standard of reference, normal values from the local population were used. To assess the nutritional status, body mass index (BMI) interpretations of OLAF percentile charts were used [12]. For children younger than 6 years, World Health Organization (WHO) growth standards were used. Underweight was defined as BMI −2 SD and lower, being overweight as BMI +1 SD, obesity +2 SD and upper percentile for age and sex [13]. The BMI value was calculated according to the formula: body mass (kg)/height (m^2^) [14]. 

### 2.3. Dietary Assessment

The quantitative assessment of the diet was made on the basis of the analysis of the children’s food diaries for three days, two weekdays, and one weekend. This method consists of carefully listing all products, meals, and drinks that are consumed during these 3 days, including a detailed description of the foods and beverages consumed, their amount (portion size), brand (if relevant), and preparation (e.g., cooking method, the addition of fat, recipe, ingredients, etc.). Data collected from each child included three food diaries at three time points: before starting the gluten-free diet and after 6 and 12 months following the GFD.

Parents received food diaries at the first visit with instructions on how to fill them in. The completed diaries were returned at the follow-up visits.

### 2.4. Blood Parameters

Biochemical and immunological laboratory blood tests were taken at the beginning and at each follow-up visit. Tests for fasting total cholesterol (CHOL; normal range: 2.95–5.57 mmol/L), high-density lipoprotein (HDL; normal range: 0.61–2.18 mmol/L), low-density lipoprotein (LDL; normal range: 1.51–3.34 mmol/L), triglycerides (TG; normal range: 0.5–2.19 mmol/L) and iron (normal range: 3.6–26 µmol/L), as well as TTG IgA, were carried out in specialized laboratories of the University Children’s Hospital in Krakow according to routine laboratory procedures designed for medical diagnosis in accordance with GPL and IVD/FDA standards.

### 2.5. Data Analysis

The Aliant calculator was used to evaluate the implementation of nutrition standards. The software used for the nutrient analysis of the food records included the Polish National Food and Nutrition Institute database. Dietary supplements were not included in the nutrient calculations because only vitamin D was supplemented in the studied group. The values of macro and micronutrients were referred to the Polish norm according to the age and sex of the child [15]. To evaluate the prevalence of nutrient adequacy, Estimated Energy Requirement (EER) for energy, Reference Intake (RI) for fats, saturated fats (SFA) and carbohydrates, Adequate Intake (AI) for monounsaturated fats (MUFA), polyunsaturated fats (PUFA) and cholesterol were used. Recommended Dietary Allowance (RDA) and AI were used for micronutrients and vitamins.

All the collected data were analyzed statistically with the use of Statistica 13.0 software (StatSoft). The Friedman test was used to compare the differences in the studied group with repeated measures. Correlations between the change in percentiles for BMI and nutrient intake were checked with the use of Spearman’s rank correlation. The significance level was set to *p* < 0.05. 

The study was conducted following the Declaration of Helsinki for medical research [16] and with the positive approval of the Jagiellonian University Bioethics Commission (No. 1072.6120.82.2018). 

## 3. Results

The study group included 40 children, 28 girls (70%) and 12 boys (30%) aged 7–17.5 years old. The mean age of the children at the beginning of the study was 8.43 years (SD 3.5). The body height changed by an average of 8 cm during the course of the year (*p* < 0.01) while body weight increased, on average, by 4kg (*p* < 0.01). There was no significant increase in BMI or BMI percentile after 6 months (accordingly Δ = 0.08, Δ = −0.42) and 12 months of the study (accordingly Δ = 0.32, Δ = −0.05), cf. Table 1.

**Figure 2:**
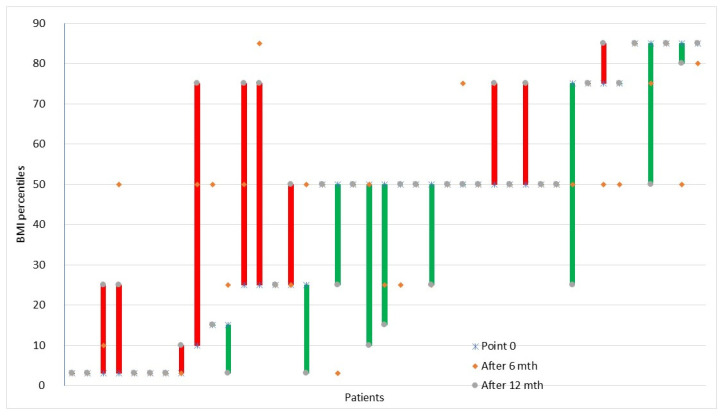
Changes in individual children’s BMI percentiles in time. The lines describe the BMI percentile change individually for each child between three time points: before diagnosis (point 0) and after 6 and 12 months. Red means increase and green means decrease.

At diagnosis, 67.5% of the children had proper BMI, 20% were underweight and 2.5% were overweight. After six months, the number of children with normal BMI increased by 7.5%, the number of underweight and overweight children decreased accordingly by 2.5% and 5%. The change in the BMI percentile for each child is presented in Figure 2. 

**Figure 3:**
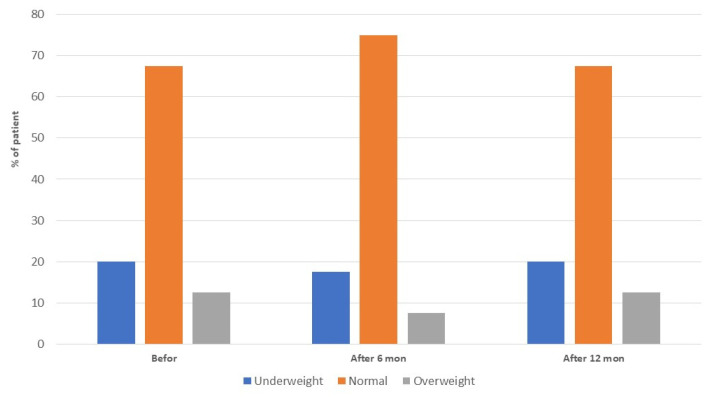
Classification of study participants according to BMI percentiles at the three time points.

After a year of the study, the percentage distribution of children in the three groups (underweight, normal, overweight) was the same as at the beginning of the study (Figure 3). There were no obese children in the study group at any point in time. 

No statistically significant changes in biochemical parameters were observed except for a significant increase in HDL cholesterol, cf. Table 2.

The average amount of energy consumed before the introduction of GFD increased by 110.70 kcal after 6 months and by 144.54 kcal after 12 months (*p* = 0.02), but the percentage of implementation EER norm did not change significantly after a half year and one year of the study, 93.60% (21.11) vs. 96.53% (96.53) vs. 95.97% (21.00); *p* = 0.73. There was no statistically significant increase in the consumption of fats, proteins, carbohydrates, or fiber during the year of using the GFD, and the implementation of the norm for the above-mentioned macronutrients did not change either. Significant changes were observed for polyunsaturated fatty acids (PUFAs) and cholesterol. The percentage distribution of energy from proteins, fats, and carbohydrates did not change significantly at subsequent time points of the analysis (Table 3).

Before diagnosis, most of the micronutrient intake followed the nutritional norms—the exception was the low and very low implementation of the dietary norm for calcium, iodine, iron, and selenium. No significant changes were observed after six months and one year of using the diet in terms of the implementation of nutritional standards for these ingredients. The percentage of implementation of the norm for zinc, iodine and potassium increased statistically in the next measurement points and were, accordingly, for zinc 114.08% (4.97) vs. 119.27% (35.84) vs. 129.51 (39.58), *p* < 0.01; iodine 13.23% (7.68) vs. 17.38% (8.77) vs. 15.79% (6.90), *p* < 0.01; potassium 119.68% (52.56) vs. 128.51% (56.75) vs. 137.88 (60.32), *p* = 0.02 (Table 4).

Concerning vitamins, low implementation of the nutritional norm was observed at the beginning of the study for folic acid and vitamins D, E, and K. After one year of using the GFD, the percentage of the implementation of nutritional standards did not change for any of the vitamins, except vitamin D, for which a statistically significant rise was observed: 11.47% (6.19) vs. 14.30% (11.58) vs. 23.90% (53.91), *p* < 0.01; however, the implementation of the norm was still very low. After six months and one year on the GFD, vitamin C intake increased significantly (Table 5). No significant correlation was observed between the change in BMI and nutrient intake percentiles.

## 4. Discussion

Celiac disease is most often associated with a deterioration of the nutritional status as most studies indicate that, at the time of diagnosis, children are underweight and shorter than their peers [8]. The elimination of the toxic factor, which is gluten, should enhance the results of absorption and, consequently, provide an improvement in the nutritional status [7]. In the study group, 22% of children at the beginning of the study were stunted according to the WHO definition; after one year, this result dropped to 15%. Statistically significant growth of height and height’s percentiles was observed [17]. 

Brambilla et al. compared 150 children under GFD from a median (IQR) time of 4.4 (4.2) years with 288 healthy children, matched for gender and age. The mean BMI of the CD patients was significantly lower than that of the healthy children in the control group. Following the introduction of a GFD, the number of children who were underweight decreased significantly and the number of those who were overweight increased [9]. In our study, the children grew significantly and increased their body weight, but not so much that there was a significant improvement in their nutritional status expressed in BMI or BMI percentiles, which may be the result of a negative energy balance. Children at the beginning and after one year of the study consumed too few calories and did not meet 100% of the Estimated Energy Requirement.

In our study, we did not find relevant differences in the nutrient value of the diet of children and adolescents before diagnosis and after one year of using GFD. According to the macronutrient distribution profile, the observed patients followed a high-lipid, high-protein, low-carbohydrate diet compared to the Polish Recommendation for lipids (less than 35% of total energy) and proteins (between 10 and 15% of total energy) and carbohydrates (between 50 and 60% of total energy) [15]. These results are consistent with the results for the general Polish population [18,19,20]. Similar results in CD patients were seen in the work of Bardella et al., in which the total energy intake was lower in celiac patients than in the control group and they consumed more energy in the form of fats compared to the levels of carbohydrates [21].

According to the dietary lipid profile, children and adolescents failed to meet the recommendations, with a higher contribution of saturated fatty acids (SFAs) and monounsaturated fatty acids (MUFAs) to total energy intake and a lower contribution of PUFAs, as compared to the guidelines. After one year of using GFD, the percentage of norm implementation for PUFAs decreased by 40% and raised by 15% for cholesterol. Regarding cholesterol intake, at any point in time, a higher intake than the recommended maximum limit of 300 mg/day was not observed. The increase in dietary fat consumption has been observed in many studies of children with CD [22,23,24,25,26,27,28]. High consumption of SFAs and cholesterol, as well as low absorption of PUFAs, are common in the pediatric population in Europe, and GFD may add an extra dose of lipids [29]. This state may be caused by the consumption of gluten-free products, to which fats are often added to improve the taste and increase the caloric value. In the study of Calvo-Lerma et al., gluten-free products (GFP) were compared with their gluten-containing counterparts (GCC); it was observed that bread (GFP) had higher total and saturated fat contents in which palm oil was the main fat used [30]. High intake of SFAs is one of the causes of dyslipidemia, as well as cardiovascular and liver diseases. The effect of GFD on nonalcoholic fatty liver disease (NAFLD) and nonalcoholic steatohepatitis (NASH) and metabolic disorders in adult individuals with CD is a matter of debate [31,32,33]. A recently published review found alterations of the lipid profile suggesting that important changes happen in celiac patients on a GFD, though the physiopathology of these conditions is unclear [34]. In our study, we did not observe significant changes in lipid profiles after one year, except for HDL, which was slightly raised.

Concerning carbohydrates, an increase in consumption by 25 g during the year of dieting was observed; however, this increase was in line with the increase in demand because no differences were observed in the implementation of nutritional norms for carbohydrates at the beginning and the end of the study, and the overall diet of children was rather low in carbohydrates.

Insufficient intake of fiber was observed at the beginning of the study; however, during the following GFD, a statistically insignificant change was observed. Gluten-free products currently have a similar fiber content [29], but vegetables and fruits are also good sources of fiber, of which intake is insufficient in the Polish children population [35]. Fiber is a prebiotic that can be metabolized by microbes in the gastrointestinal tract and modulate the microbiota. Studies by Cenit et al. have shown that patients with CD have a reduced number of *Bifidobacterium* spp. and an increased number of *Bacteroides* spp. Changes in the intestinal microbiota, however, are not completely normalized after the introduction of GFD. Even a two-year GFD does not restore the composition of the bacterial flora to the physiological state [36]. In other studies, the concentration of *Bifidobacterium*, *Enterococcus*, and *Lactobacillus* and an increase in *Klebsiella*, *Salmonella*, *Bacteroides*, *Staphylococcus*, and *Shigella* concentrations were shown in celiac patients [37,38]. Additionally, in the case of a smaller number of Gram-positive bacteria, the colony of *Bacteroides fragilis* may grow, which releases proteolytic enzymes causing the formation of toxic proteins from gluten [39].

The results of studies from different countries in different age groups have consistently shown that celiac patients are at risk of various nutritional deficiencies. These deficiencies are largely the result of damage to the intestinal mucosa and malabsorption, but during the course of GFD, deficiencies are mainly caused by an improperly balanced diet. Dietary complications in patients with CD on GFD can be caused by the poor nutritional value of commercial gluten-free products, and a complicated selection of food products. Deficiencies seen in celiac patients using GFD include fiber, iron, folic acid, niacin, vitamin B12, and riboflavin [40,41]. In our study, we analyzed the adherence of actual food intake to the recommended consumption for these nutrients. In children and adolescents with CD, we found adequate consumption concerning the norms for most water-soluble vitamins, i.e., thiamine, riboflavin, pyridoxine, niacin, cobalamin and vitamin C. For these vitamins, no change was observed during the year of following the GFD. Thus, there is no risk of deficiency of these vitamins, at least in our study group. A much bigger problem is the lack of adequacy of the nutritional norm for folic acid and fat-soluble vitamins such as D, E, and K. Low intake of folic acid and vitamin K corresponds to low consumption of vegetables, especially green ones, as indicated by studies by other authors, but this trend applies to the entire pediatric population [35].

Vitamin D intake from food products was much lower than the recommendations for children and adolescents, which is consistent with the results in the general population regarding vitamin D deficiency [42]. The main source of vitamin D is radial skin biosynthesis, not a diet. Due to the common lack of this vitamin in the pediatric population, all children should be supplemented as recommended [43]. In our study group, all children actually used vitamin D3 supplementation.

As shown by the results of studies in the population of Polish children, chronic calcium deficiency is present in the diet of 51% of children [35,42]. Very low calcium intake among children with celiac disease was observed in our study. This finding is also confirmed by the results of other authors [41,44]. Vitamin D and calcium play a crucial role in proper bone mineral density (BMD). Another important vitamin linked to the risk of bone disorders is vitamin K2. It interacts with calcium and vitamin D and is essential for the activation of osteocalcin. Moreover, vitamin K2 has been shown to inhibit bone resorption by suppression of the prostaglandin E2 synthesis in osteoclasts [45,46]. Children have the highest need for vitamin K since bone formation and development are most intense during childhood and adolescence. Krzesiek et al. observed a reduction in BMD in 40% of children diagnosed with CD and in 75% of patients with newly diagnosed CD [47]. With regard to these results and nutritional deficiency, there is a need for early assessment of bone mineral density (BMD) to treat bone abnormalities in celiac patients since bone disorders are well documented [48,49,50]. Perhaps mandatory supplementation of GFD with calcium and vitamin K2, not only vitamin D, needs to be considered for such patients.

Low adherence to the iron norm concerning low serum iron levels is worrying, especially since there has been no significant increase in iron intake and adequate increase in serum iron levels during the year of the diet. The children in our study were not supplemented with iron, so this result reflects the real level of intake. In the studies of other authors, too low iron intake is also observed and, if we take into account the fact that people with celiac disease often develop anemia, this nutrient should be under special control [39].

## 5. Conclusions

The results of our study revealed that there is no risk of deficiency of water-soluble vitamins, except for folic acid, but special attention should be given to the correct supply of vitamin D, K and E. The low intake of key nutrients for child development, such as calcium, iron and iodine, observed before the diagnosis, did not improve after the introduction of GFD. There was also no significant change in the implementation of the norm for energy in children following GFD. Taking into account the increased energy needs due to inflammation and the healing process, some CD children on GFD may have a negative energy balance. These results confirm the need for personalized nutritional education focused not only on excluding gluten from the diet but also reducing nutritional deficiencies by selecting foods rich in nutrients when a deficiency in the diet is observed. If integration with the diet is not enough, administration of supplements may be the correct way, after evaluating the initial blood level to determine the right dosage of supplementation. The strength of this study is that the changes were assessed over time by analyzing the children’s diet, anthropometric and biochemical parameters at three time points. Regarding the weaknesses, the method of food tracking (food diary) has a limitation in the form of an underestimated or overestimated portion size by the respondent, which may distort the actual nutrient intake. The second limitation is that the follow-up of the patients of the study was only one year.

## Figures and Tables

**Table 1:** Changes of anthropometric parameters in the studied group.

Variables	Before Diet X (SD)	After 6 Mon X (SD)	After 12 Mon X (SD)	*p*-Value
Weight (kg)	28.15 (2.64)	29.76 (2.38) Δ = 1.61	32.34 (13.50) Δ = 4.19	0.01
Weight percentile	39.85 (8.61)	45.13 (33.66) Δ = 5.48	47.68 (32.38) Δ = 8.03	0.01
Height (cm)	127.88 (18.94)	131.39 (18.12) Δ = 3.51	135.90 (17.90) Δ = 8.03	0.01
Height percentile	39.65 (32.18)	45.13 (33.66) Δ = 5.48	47.68 (47.68) Δ = 8.03	0.01
BMI (kg/m^2^)	16.36 (2.60)	16.44 (2.47) Δ = 0.08	16.68 (2.78) Δ = 0.32	0.15
BMI percentile	42.23 (27.93)	42.65 (25.38) Δ = 0.42	42.28 (28.66) Δ = −0.05	0.95

X—mean; SD values due to normal distribution; Δ = change value; a *p*-value of less than 0.05 was considered significant.

**Table 2:** Changes of biochemical parameters in the studied group.

Variables	Before Diet X (SD)	After 6 Mon X (SD)	After 12 Mon X (SD)	*p*-Value *
CHOL (mmol/L)	4.06 (0.68)	4.29 (0.93) Δ = 0.23	4.30 (0.85) Δ = 0.24	0.04
HDL (mmol/L)	1.37 (0.32)	1.59 (0.42) Δ = 2.22	1.53 (0.41) Δ = 0.16	<0.01
LDL (mmol/L)	2.40 (0.56)	2.50 (0.80) Δ = 0.1	2.43 (0.77) Δ = 0.33	0.98
TG (mmol/L)	0.83 (0.37)	0.67 (0.21) Δ = −0.16	0.73 (0.30) Δ = −0.1	0.15
Fe (µmol/L)	15.90 (6.99)	17.37 (6.63) Δ = 1.47	17.20 (6.07) Δ = 1.30	0.56

X—mean; SD values due to normal distribution; Δ = change value; a *p*-value of less than 0.05 was considered significant, * *p*-value referred to before diet and after 12 months; CHOL—Total Cholesterol; HDL—High-Density Lipoprotein; LDL—Low-Density Lipoprotein; TG—Triglycerides; Fe—Iron.

**Table 3:** Daily dietary intake and norm implementation of energy, macronutrients and fiber in the studied group.

Intake	Before X (SD)	After 6 Mon X (SD)	After 12 Mon X (SD)	*p*-Value *
Energy kcal/day	1440.59 (367.44)	1551.30 (384.14) Δ = 110.71	1629.11 (368.09) Δ = 188.52	0.02
Energy % EER	93.60 (21.11)	96.53 (22.53) Δ = 3.38	95.97(21.00) Δ = 2.38	0.73
Protein g/day	58.42 (16.04)	61.80 (16.47) Δ = 6.2	64.03 (13.90) Δ = 5.62	0.43
Protein % RDA	111.17 (45.29)	105.22 (31.79) Δ = −5.95	105.54 (30.21) Δ = −5.63	0.55
Fats g/day	54.83 (18.56)	58.97 (18.63) Δ = 4.14	62.47 (17.99) Δ = 7.63	0.19
Fats % RI	106.57 (31.82)	111.15 (38.96) Δ = 4.58	110.62 (30.30) Δ = 4.06	0.79
SFA g/day	20.80 (7.90)	23.31 (8.14) Δ = 2.51	24.57 (7.57) Δ = 3.77	0.28
SFA % RI	160.39 (66.39)	179.60 (86.90) Δ = 19.21	184.40 (86.13) Δ = 24.01	0.79
MUFA g/day	20.93 (8.04)	22.43 (7.84) Δ = 1.51	24.16 (7.47) Δ = 3.23	0.10
MUFA % AI	125.04 (61.40)	122.33 (55.29) Δ = −2.71	121.47 (43.17) Δ = −3.57	0.79
PUFA g/day	14.68 (29.89)	11.64 (26.17) Δ = −3.04	8.39 (3.63) Δ = −6.30	0.04
PUFA % AI	83.38 (172.95)	75.46 (222.64) Δ = −7.92	42.93 (17.38) Δ = −40.45	0.01
Cholesterol	232.09 (95.68)	275.86 (123.56) Δ = 43.77	281.93 (94.71) Δ = 49.83	0.16
Cholesterol % AI	77.36 (31.89)	91.96 (41.18) Δ = 14.60	93.97 (31.57) Δ = 16.61	0.04
Carbohydrates g/day	189.65 (52.35)	204.23 (59.71) Δ = 14.58	214.92 (51.68) Δ = 25.27	0.01
Carbohydrates % RI	86.12 (21.86)	88.68 (27.22) Δ = 2.56	88.21 (20.10) Δ = 2.09	0.37
Fiber g/day	14.39 (5.91)	15.08 (6.86) Δ = 0.70	15.87 (4.41) Δ = 1.49	0.06
Fiber % AI	86.92 (35.41)	90.99 (41.61) Δ = 4.07	98.63 (31.80) Δ = 11.70	0.06
% Energy from Protein	16.22 (2.33)	16.06 (2.59) Δ = −13.35	15.72 (1.85) Δ = −13.74	0.56
% Energy from Fats	33.40 (7.26)	33.90 (6.54) Δ = 1.18	34.11 (5.16) Δ = 1.39	0.60
% Energy from Carbohydrates	50.22 (7.63)	50.59 (7.03) Δ = 0.51	50.50 (5.22) Δ = 0.42	0.73

X—mean; SD values due to normal distribution. Δ = change value; a *p*-value of less than 0.05 was considered significant. * *p*-value referred to before diet and after 12 months; % EER—Percentage of implementation of Estimated Energy Requirement norm; % RDA—Percentage of implementation of Recommended Dietary Allowance norm; % RI—Percentage of implementation of Reference Intake; % AI—Percentage of implementation of Adequate Intake norm; SFA—Saturated Fats; MUFA—Monounsaturated Fats; PUFA—Polyunsaturated Fats.

**Table 4:** Daily dietary intake and norm implementation of micronutrients in the studied group.

Intake	Before X (SD)	After 6 Mon X (SD)	After 12 Mon X (SD)	*p*-Value *
Sodium (mg)	1541.7 (615.6)	1732.0 (679.4) Δ = 190	1725.6 (618.45) Δ = 183	0.56
% AI	127.89 (49.13)	145.08 (56.70) Δ = 17.2	143.12 (45.78) Δ = 15.2	0.56
Potassium (mg)	2055.26 (687.85)	2901.67 (685.63) Δ = 135.4	2335.84 (568.94) Δ = 280	0.03
% AI	119.68 (6.19)	128.51 (56.75) Δ = 8.80	137.88 (60.32) Δ = 18	0.02
Calcium (mg)	537.13 (233.32)	616.16 (242.07) Δ = 80.0	547.85 (202.03) Δ = 10.72	0.43
% RDA	50.56 (23.60)	57.17 (21.59) Δ = 6.60	50.72 (18.76) Δ = 0.15	0.43
Phosphorus (mg)	912.98 (290.83)	990.64 (277.64) Δ = 77.67	1015.39 (233.23) Δ = 102.41	0.26
% RDA	136.98 (61.84)	147.30 (57.28) Δ = 0.29	149.54 (57.92) Δ = 12.56	0.37
Magnesium (mg)	197.27 (71.20)	214.01 (68.85) Δ = 16.74	215.53 (49.49) Δ = 18.26	0.52
% RDA	126.92 (69.23)	135.31 (57.75) Δ = 8.39	135.04 (50.45) Δ = 8.12	0.65
Iron (mg)	8.40 (3.01)	9.49 (4.65) Δ = 1.10	7.97 (1.69) Δ = −4.43	0.17
% RDA	79.29 (38.82)	90.55 (50.88) Δ = 11.26	75.03 (32.76) Δ = −4.26	0.25
Zinc (mg)	6.5 (0.92)	6.86 (1.75) Δ = 0.35	7.48 (1.57) Δ = 0.97	<0.01
% RDA	114.08 (44.97)	119.27 (35.84) Δ = 5.19	129.51 (39.58) Δ = 15.44	<0.01
Copper (mg)	0.77 (0.32)	0.80 (0.28) Δ = 0.03	0.86 (0.24) Δ = 0.09	0.08
% RDA	129.90 (67.77)	122.07 (59.28) Δ = 3.17	148.59 (68.40) Δ = 18.69	0.11
Manganese (mg)	2.53 (1.27)	2.20 (1.52) Δ = −0.33	2.04 (0.76) Δ = −0.49	0.66
% AI	163.66 (82.49)	141.38 (97.93) Δ = −22.28	130.90 (50.92) Δ = −32.76	0.73
Selenium (ug)	6.04 (7.62)	6.76 (6.81) Δ = 0.72	5.42 (5.03) Δ = −0.63	0.66
% RDA	18.86 (25.26)	19.28 (18.72) Δ = 0.43	15.71 (15.10) Δ = −3.14	0.58
Iodine (ug)	13.57 (6.74)	18.08 (8.20) Δ = 4.53	16.53 (6.84) Δ = 2.96	<0.01
% RDA	13.23 (7.68)	17.38 (11.58) Δ = 4.16	15.79 (6.90) Δ = 2.57	<0.01

X—mean; SD values due to normal distribution; Δ = change value; a *p*-value of less than 0.05 was considered significant. * *p*-value referred to before diet and after 12 months; % RDA—Percentage of implementation of Recommended Dietary Allowance norm; % AI—Percentage of implementation of Adequate Intake norm.

**Table 5:** Daily dietary intake and norm implementation of vitamins in the studied group.

Intake	Before X (SD)	After 6 Mon X (SD)	After 12 Mon X (SD)	*p*-Value *
Vit. A (ug)	671.91 (377.25)	757.52 (349.38) Δ = 85.61	778.96 (350.44) Δ = 107.5	0.11
% RDA	131.69 (84.99)	146.72 (75.13) Δ = 15.03	156.14 (96.25) Δ = 24.45	0.11
Vit. D (ug)	1.72 (0.92)	2.15 (1.74) Δ = 0.43	3.59 (8.09) Δ = 1.88	<0.01
% AI	11.47 (6.19)	14.30 (11.58) Δ = 2.83	23.90 (53.91) Δ = 12.43	<0.01
Vit. E (mg)	6.15 (2.98)	6.29 (3.05) Δ = 0.05	6.64 (2.94) Δ = 0.50	0.25
% AI	87.01 (41.68)	88.26 (44.46) Δ = 1.25	93.63 (42.99) Δ = 6.62	0.41
Vit. K (ug)	4.88 (7.59)	4.13 (6.33) Δ = −0.75	4.20 (4.75) Δ = −0.68	0.71
% AI	8.43 (13.50)	7.11(10.72) Δ = −1.32	7.23 (8.04) Δ = −1.20	0.79
Vit. B1 (mg)	1.00 (0.48)	1.05 (0.41) Δ = 0.05	1.15 (1.06) Δ = 0.15	0.72
% RDA	116.48 (49.18)	128.47 (61.85) Δ = 11.99	141.21 (153.78) Δ = 24.73	0.76
Vit. B2 (mg)	1.28 (0.50)	1.50 (0.69) Δ = 0.22	1.36 (0.37) Δ = 0.07	0.46
% RDA	151 (58.42)	183 (109.41) Δ = 31.72	163.91 (59.11) Δ = 12.52	0.34
Vit. B3 (mg)	13.03 (5.54)	15.31 (6.63) Δ = 2.99	14.41 (3.86) Δ = 1.38	0.23
% RDA	118.43 (48.34)	143.84 (76.67) Δ = 25.41	134.16 (44.89) Δ = 15.74	0.23
Vit. B6 (mg)	1.49 (0.49)	1.73 (0.76) Δ = 0.24	1.62 (0.41) Δ = 0.13	0.61
% RDA	164.25 (62.34)	200.57 (121.88) Δ = 36.32	184.80 (74.62) Δ = 20.56	0.60
Folic acid (ug)	160.96 (60.03)	193.85 (83.69) Δ = 32.90	188.89 (51.52) Δ = 27.93	0.21
% RDA	58.20 (24.26)	71.76 (39.96) Δ = 13.56	70.03 (26.80) Δ = 11.83	0.21
Vit. B12 (mg)	2.53 (1.07)	2.96 (1.17) Δ = 0.44	2.97 (1.37) Δ = 0.44	0.09
% RDA	153.10 (72.94)	182.50 (90.06) Δ = 29.41	180.05 (90.02) Δ = 26.95	0.08
Vit. C (mg)	63.92 (36.48)	81.70 (53.98) Δ = 17.79	93.21 (55.10) Δ = 29.29	<0.01
% RDA	123.80 (75.58)	156.77 (102.89) Δ = 32.98	179.73 (122.25) Δ = 55.94	<0.01

X—mean; SD values due to normal distribution; Δ = change value; a *p*-value of less than 0.05 was considered significant. * *p*-value referred to before diet and 12 month; % RDA—Percentage of implementation of Recommended Dietary Allowance norm; % AI—Percentage of implementation of Adequate Intake norm.

## Data Availability

The data presented in this study are not publicly available due to confidentiality reasons. These data are available on request from the corresponding author.

## References

[1] Kaukinen K., Partanen J., Mäki M., Collin P. (2002). HLA-DQ typing in the diagnosis of celiac disease. Am. J. Gastroenterol..

[2] Husby S., Koletzko S., Korponay-Szabó I.R., Mearin M.L., Phillips A., Shamir R., Troncone R., Giersiepen K., Branski D., Catassi C. (2012). for the ESPGHAN Working Group on Coeliac Disease Diagnosis, on behalf of the ESPGHAN Gastroenterology Committee European Society for Pediatric Gastroenterology, Hepatology, and Nutrition Guidelines for the Diagnosis of Coeliac Disease. J. Pediatr. Gastroenterol. Nutr..

[3] Al-Toma A., Volta U., Auricchio R., Castillejo G., Sanders D.S., Cellier C., Mulder C.J., Lundin K.E.A. (2019). European Society for the Study of Coeliac Disease (ESsCD) guideline for coeliac disease and other gluten-related disorders. United Eur. Gastroenterol. J..

[4] Singh P., Arora A., Strand T.A., Leffler D.A., Catassi C., Green P.H., Kelly C.P., Ahuja V., Makharia G.K. (2018). Global Prevalence of Celiac Disease: Systematic Review and Meta-analysis. Clin. Gastroenterol. Hepatol..

[5] Bascuñán K.A., Roncoroni L., Branchi F., Doneda L., Scricciolo A., Ferretti F., Aray M., Elli L. (2018). The 5 Ws of a gluten challenge for gluten-related disorders. Nutr. Rev..

[6] Grzymisławski M., Stankowiak-Kulpa H., Włochal M. (2010). Celiakia—Standardy diagnostyczne i terapeutyczne 2010 roku. Forum Zaburzeń Metab..

[7] Wierdsma N.J., Berkenpas M., Mulder C.J.J., Van Bodegraven A.A. (2013). Vitamin and Mineral Deficiencies Are Highly Prevalent in Newly Diagnosed Celiac Disease Patients. Nutrients.

[8] van der Pals M., Myléus A., Norström F., Hammarroth S., Högberg L., Rosén A., Ivarsson A., Carlsson A. (2014). Body mass index is not a reliable tool in predicting celiac disease in children. BMC Pediatr..

[9] Brambilla P., Picca M., Dilillo D., Meneghin F., Cravidi C., Tischer M.C., Vivaldo T., Bedogni G., Zuccotti G.V. (2013). Changes of body mass index in celiac children on a gluten-free diet. Nutr. Metab. Cardiovasc. Dis..

[10] Reilly N.R., Aguilar K., Hassid B.G., Cheng J., Defelice A.R., Kazlow P., Bhagat G., Green P.H. (2011). Celiac disease in normal-weight and overweight children: Clinical features and growth outcomes following a gluten-free diet. J. Pediatr. Gastroenterol. Nutr..

[11] Oberhuber G., Granditsch G., Vogelsang H. (1999). The histopathology of coeliac disease: Time for a standardized report scheme for pathologists. Eur. J. Gastroenterol. Hepatol..

[12] de Onis M., WHO Multicentre Growth Reference Study Group (2006). Assessment of differences in linear growth among populations in the WHO Multicentre Growth Reference Study. Acta Paediatr..

[13] Kułaga Z., Litwin M., Tkaczyk M. (2011). Polish 2010 growth references for school-aged children and adolescents. Eur. J. Pediatr..

[14] Slaughter M.H., Lohman T.G., Boileau R., Horswill C.A., Stillman R.J., Van Loan M.D., Bemben D.A. (1988). Skinfold equations for estimation of body fatness in children and youth. Hum. Boil..

[15] Jarosz M., Rychlik E., Stoś K., Charzewska J. (2020). Nutrition Standards for the Polish Population and Their Applied.

[16] Puri K.S., Suresh K.R., Gogtay N.J., Thatte U.M. (2009). Declaration of Helsinki, 2008: Implications for stakeholders in research. J. Postgrad. Med..

[17] WHO (2014). Global Nutrition Targets 2025: Stunting Policy Brief.

[18] Falkowska A., Stefańska E., Ostrowska L. (2011). Assessment of the diet of children aged 10–12 with various levels of nutrition. Endokrynol. Otył. Zab. Przem. Mat..

[19] Merkiel-Pawłowska S., Chalcarz W. (2017). Gender differences and typical nutrition concerns of the diets of preschool children—The results of the first stage of an intervention study. BMC Pediatr..

[20] Florkiewicz A., Grzych-Tuleja E., Cieślik E., Topolska K., Filipiak-Florkiewicz A., Leszczyńska T., Kopeć A. (2013). Assessment of dietary intake of selected minerals by adolescents aged 13–15 years depending on gender and place of residence. Zdr. Publ. Zarządz..

[21] Bardella M.T., Fredella C., Prampolini L., Molteni N., Maria Giunta A., Bianchi A.A. (2000). Body Composition and Dietary Intakes in Adult Celiac Disease Patients Consuming a Strict Gluten-Free Diet. Am. J. Clin. Nutr..

[22] Mariani P., Viti M.G., Montouri M., La Vecchia A., Cipolletta E., Calvani L., Bonamico M. (1998). The gluten free diet: A nutritional risk factor for adolescents with celiac disease?. J. Pediatr. Gastroenterol. Nutr..

[23] Balamtekin N., Aksoy C., Baysoy G., Uslu N., Demir H., Koksal G., Saltık-Temizel I.N., Özen H., Gürakan F., Yüce A. (2015). Is compliance with gluten-free diet sufficient? Diet composition of celiac patients. Turk. J. Pediatr..

[24] Babio N., Alcázar M., Castillejo G., Recasens M., Martínez-Cerezo F., Gutiérrez-Pensado V., Masip G., Vaqué C., Vila-Martí A., Torres-Moreno M. (2017). Patients with celiac disease reported higher consumption of added sugar and total fat than healthy individuals. J. Pediatr. Gastroenterol. Nutr..

[25] Rujner R., Socha J., Syczewska M., Wojtasik A., Stolarczyk A. (2004). Magnesium Status in Children and Adolescents with Coeliac Disease Without Malabsorption Symptoms. Clin. Nutr..

[26] Alzaben A.S., Turner J., Shirton L., Tarah M.S., Rabin P., Mager D. (2015). Assessing nutritional quality and adherence to the gluten-free diet in children and adolescents with celiac disease. Can. J. Diet. Pr. Res..

[27] Sue A., Dehlsen K., Ooi C.Y. (2018). Pediatric Patients with Coeliac Disease on a Gluten-Free Diet: Nutritional Adequacy and Macro- and Micronutrient Imbalances. Curr. Gastroenterol. Rep..

[28] Larretxi I., Simon E., Benjumea L., Miranda J., Bustamante M.A., Lasa A., Eizaguirre F.J., Churruca I. (2018). Gluten-free-rendered products contribute to imbalanced diets in children and adolescents with celiac disease. Eur. J. Nutr..

[29] Ruiz E., Ávila J., Valero T., Del Pozo S., Rodriguez P., Aranceta-Bartrina J., Gil Á., González-Gross M., Ortega R., Serra-Majem L. (2016). Macronutrient Distribution and Dietary Sources in the Spanish Population: Findings from the ANIBES Study. Nutrients.

[30] Calvo-Lerma J., Crespo-Escobar P., Martínez-Barona S., Fornés-Ferrer V., Ribes-Koninckx C. (2019). Differences in the macronutrient and dietary fibre profile of gluten-free products as compared to their gluten-containing counterparts. Eur. J. Clin. Nutr..

[31] Tortora R., Capone P., De Stefano G., Imperatore N., Gerbino N., Donetto S., Monaco V., Caporaso N., Rispo A. (2015). Metabolic syndrome in patients with coeliac disease on a gluten-free diet. Aliment. Pharmacol. Ther..

[32] Ciccone A., Gabrieli D., Cardinale R., di Ruscico M. (2018). Metabolic Alterations in Celiac Disease Occurring after Following a Gluten-Free. Diet. Dig..

[33] García-Manzanares A., Lucendo A.J., González-Castillo S., Moreno-Fernández J. (2011). Resolution of metabolic syndrome after following a gluten free diet in an adult woman diagnosed with celiac disease. World J. Gastrointest. Pathophysiol..

[34] Valvano M., Longo S., Stefanelli G., Frieri G., Viscido A., Latella G. (2020). Celiac Disease, Gluten-Free Diet, and Metabolic and Liver Disorders. Nutrients.

[35] Mazur J., Małkowska-Szkutnik A. (2018). Student Health in 2018 against the Background of a New Research Model HBSC.

[36] Cenit M.C., Olivares M., Codoñer-Franch P., Sanz Y. (2015). Intestinal microbiota and celiac disease: Cause, consequence or co-evolution?. Nutrients.

[37] Di Cagno R., De Angelis M., De Pasquale I., Ndagijimana M., Vernocchi P., Ricciuti P., Gagliardi F., Laghi L., Crecchio C., Guerzoniet M.E. (2011). Duodenal and faecal microbiota of celiac children: Molecular, phenotype and metabolome characterization. BMC Microbiol..

[38] Cenit M.C., Codoñer-Franch P., Sanz Y. (2016). Gut microbiota and risk of developing celiac disease. J. Clin. Gastroenterol..

[39] Sánchez E., Laparra J.M., Sanz Y. (2012). Discerning the role of Bacteroides fragilis in celiac disease pathogenesis. Appl. Environ. Microbiol..

[40] Penagini F., DiLillo D., Meneghin F., Mameli C., Fabiano V., Zuccotti G.V. (2013). Gluten-Free Diet in Children: An Approach to a Nutritionally Adequate and Balanced Diet. Nutrients.

[41] Ballestero Fernández C., Varela-Moreiras G., Úbeda N., Alonso-Aperte E. (2019). Nutritional Status in Spanish Children and Adolescents with Celiac Disease on a Gluten Free Diet Compared to Non-Celiac Disease Controls. Nutrients.

[42] Weker H., Barańska M., Riahi A., Strucińska M., Więch M., Rowicka G., Dyląg H., Klemarczyk W., Bzkowska A., Socha P. (2017). Dietary patterns in toddlers with excess weight. The 2016 PITNUTS study. Dev. Period Med..

[43] Rusińska A., Płudowski P., Walczak M., Borszewska-Kornacka M.K., Bossowski A., Chlebna-Sokół D., Justyna Czech-Kowalska J., Dobrzańska D., Franek E. (2018). Vitamin D supplementation guidelines for Poland—2018 update. Stand. Med. Pediatr..

[44] Krupa-Kozak U. (2014). Pathologic bone alterations in celiac disease: Etiology, epidemiology, and treatment. Nutrition.

[45] Weber P. (1997). Management of osteoporosis: Is there a role for vitamin K?. Int. J. Vitam. Nutr. Res..

[46] Gordeladze J.O., Landin M.A., Johnsen G.F., Haugen H.J., Osmundsen H. (2017). Vitamin K2 and its Impact on Tooth Epigenetics. Vitamin K2—Vital for Health and Wellbeing.

[47] Krzesiek E., Iwańczak B. (2008). Assessment of bone mineral density in children with celiac disease. Pol. Merkur. Lek. Organ. Pol. Tow. Lek..

[48] Motta M.E.F.A., De Faria M.E.N., Da Silva G.A.P. (2009). Prevalence of low bone mineral density in children and adolescents with celiac disease under treatment. Sao Paulo Med. J..

[49] Vázquez H., Mazure R., Gonzalez D., Flores D., Pedreira S., Niveloni S., Smecuol E., Mauriño E., Bai J.C. (2000). Risk of fractures in celiac disease patients: A cross-sectional, case-control study. Am. J. Gastroenterol..

[50] Bianchi M.L., Bardella M.T. (2008). Bone in celiac disease. Osteoporos. Int..

